# Time-resolved X-ray diffraction studies of mineral transformations in aqueous solutions

**DOI:** 10.1063/4.0000940

**Published:** 2025-10-27

**Authors:** Dong Youn Chung, Peter J. Heaney, Joanne E. Stubbs, Peter J. Eng

**Affiliations:** 1 Pennsylvania State University, University Park, PA, USA; 2 The University of Chicago, Chicago, IL, USA

## Abstract

The time-resolved X-ray diffraction (TRXRD) technique enables in-situ studies of mineral transformations in aqueous solutions with a time resolution of ∼30 seconds. Rietveld analysis of sequential XRD data reveals the real-time evolution of phase fractions and crystal structure parameters of mineral transformations, as is especially important when the transformation occurs through metastable intermediate phases (i.e., by the Ostwald step rule). In contrast to traditional batch experiments, the high temporal resolution of TRXRD allows detailed observations of metastable intermediate phases, the calculation of high-quality kinetic data, and the determination of reaction mechanisms.

Two examples of the successful application of TRXRD will be discussed: the hydration of periclase (MgO) to brucite (Mg(OH)_2_), and the transformation of calcite (CaCO_3_) to dolomite (CaMg(CO_3_)_2_). In order to determine whether the periclase transforms to brucite by solid-state diffusion or by dissolution-reprecipitation, synthetic periclase powders were placed in quartz glass capillaries in an acetic acid buffer solution with a pH of 4.96 and heated to 40 to 60 °C. TRXRD data revealed a continuous increase in the unit-cell *c* parameter for brucite*.* The microstrain along the *c*-direction initially was high but decreased as a function of reaction time. Our structure refinements suggest that stacking faults generated by the propagation of hydration on the (111) plane of periclase induced the transformation to brucite.

The transformation of calcite to dolomite occurs via an intermediate phase: calcite (*S.G.*: *R-3c*) → disordered protodolomite (*S.G*: *R-3c)* → ordered dolomite (*S.G*: *R-3*). TRXRD of this reaction showed for the first time that protodolomite first crystallizes as a Mg-rich phase (∼60 mol% Mg) but evolves to a Ca-rich composition (∼60 mol%Ca). Moreover, the formation of ordered dolomite is inhibited by the presence of calcite. Both stages of dolomitization occur by dissolution and reprecipitation rather than solid-state cation exchange.

